# The correlation between reactive hyperemia index and endothelial dysfunction markers in patients with hypertension and obstructive sleep apnea syndrome: a cross-sectional study

**DOI:** 10.3389/fcvm.2026.1614324

**Published:** 2026-01-23

**Authors:** Chaoping Yu, Yue Liu, Fengcheng Xu, Bo Li, Bin Ge, Rong Zhu, Tianhu Liu, Hongyu Wang, Ying Huang, Jing Yang, Bo Zhang

**Affiliations:** 1 Department of Cardiology, The Third Affiliated Hospital of Chengdu Medical College, Chengdu Pidu District People’s Hospital, Chengdu, China; 2 Cardiology and Vascular Health Research Center of Chengdu Medical College, Chengdu, China; 3 Department of Clinical Pharmacy, The Third Affiliated Hospital of Chengdu Medical College, Chengdu Pidu District People’s Hospital, Chengdu, China; 4 Department of Laboratory Medicine, The Third Affiliated Hospital of Chengdu Medical College, Chengdu Pidu District People’s Hospital, Chengdu, China; 5 Department of Scientific Research and Teaching, The Third Affiliated Hospital of Chengdu Medical College, Chengdu Pidu District People’s Hospital, Chengdu, China; 6 Department of Vascular Medicine, Peking University Shougang Hospital, Beijing, China

**Keywords:** apnea-hypopnea index, hypertension, obstructive sleep apnea-hypopnea syndrome, reactive hyperemia index, vascular endothelial function

## Abstract

**Objective:**

Currently, there is a lack of clinical studies on how to stratify endothelial dysfunction based on the severity of co-existing hypertension and OSAHS. This evidence gap hinders clinicians’ ability to accurately assess disease burden and determine the best timing and intensity of intervention for these high-risk patients. This study aimed to investigate the impact of hypertension combined with OSAHS on vascular endothelial function.

**Methods:**

Patients aged 35–60 years with hypertension and OSAHS were consecutively recruited from the outpatient department of the Department of Cardiology at the Chengdu Pidu District People's Hospital, from July 1, 2023, to December 31, 2023. AHI, RHI and endothelial damage-related markers [Von Willebrand Factor (VWF), Vascular Endothelial Growth Factor (VEGF), and Endothelial Microparticles (EMPs)] were measured. Routine examination data were collected.

**Results:**

The correlation analysis between AHI, RHI, and hypertension grade and hypertension stage showed correlation coefficients less than 0.2, indicating almost no linear relationship. The correlation coefficient between AHI and RHI was −0.58 (*P* < 0.001). The correlation coefficients between AHI and VWF, VEGF, and EMPS were 0.56 (*P* < 0.001), 0.49 (*P* < 0.001), and 0.66 (*P* < 0.001). The correlation coefficients between RHI and VWF, VEGF, and EMPS were −0.62 (*P* < 0.001), −0.63 (*P* < 0.001), and −0.67 (*P* < 0.001). The RHI showed significant inverse associations with the studied variables.A 1-SD increase in AHI, vWF, VEGF, and EMPs was associated with a decrease in RHI of 0.02, 0.62, 0.63, and 0.67 units, respectively. (*β* = –0.02, adjusted *β* = –0.60, *P* < 0.01; *β* = –0.62, adjusted *β* = –0.64, *P* < 0.01; *β* = −0.63, adjusted *β* = –0.64, *P* < 0.01; *β* = −0.67, adjusted *β* = –0.71, *P* < 0.01).

**Conclusion:**

In patients with hypertension combined with OSAHS, RHI can be used as an important indicator in routine tests of vascular endothelial function to predict the degree of vascular endothelial injury.

## 1 Background

The “China Cardiovascular Disease Report 2023 (Summary)” indicates that the prevalence and mortality of cardiovascular diseases in China are still on the rise. Cardiovascular diseases remain the leading cause of death in China. Endothelial dysfunction is the foundation for the development of cardiovascular diseases. Therefore, early detection and intervention of endothelial damage are particularly important.

The development of cardiovascular diseases originates from endothelial dysfunction, which leads to atherosclerosis. Understanding the extent of endothelial dysfunction is crucial for intervention in the prevention of cardiovascular diseases. The status of endothelial function can be assessed through the detection of endothelial damage-related markers and invasive or non-invasive vascular examination techniques. From a clinical practice perspective, invasive techniques, such as the intracoronary injection of acetylcholine to evaluate coronary endothelial function, are highly reliable but not easily implemented routinely in clinical practice (1). Detection of endothelial damage-related markers aids in the assessment of endothelial health status (2–5), but it requires high precision in testing, which can be challenging for clinical laboratory technicians to perform accurately. Currently, non-invasive endothelial function detection techniques are gradually being adopted in clinical practice. For instance, the ultrasound-based detection of brachial artery flow-mediated dilation (FMD) demands high skill levels from operators and is challenging to widely promote in clinical settings (6). In recent years, the introduction of the Israeli peripheral arterial tonometry (PAT) technology (Endo-PAT 2000) in China has enabled the detection of the reactive hyperemia index (RHI) (7). Apart from FMD, RHI is the only non-invasive vascular endothelial function detection technique certified by the U.S. FDA (8). It is highly reproducible, and the operation process of the device is simple and easy to perform, making it more suitable for routine clinical screening.

From a functional perspective, endothelial damage-related markers and vascular examination techniques can reflect endothelial health status from multiple aspects. Von Willebrand Factor(vWF), as a structural biomarker, mainly reflects the activation and damage degree of endothelial cells, indicating impaired vascular wall integrity and increased risk of thrombosis (9, 10). Vascular Endothelial Growth Factor(VEGF), as a pro-angiogenic factor, its level changes reflect the body's adaptive response (angiogenesis and repair) to hypoxia or injury (11, 12). Endothelial Microparticles(EMPs) are vesicles shed from endothelial cells, which can serve as direct markers of endothelial injury and apoptosis, and may also act as carriers of intercellular information transmission, participating in the pathophysiological process of inflammation and vascular dysfunction (13). The reactive hyperemia index (RHI) directly reflects the dilating capacity of microvascular endothelium, especially the NO-dependent response, and is a sensitive and non-invasive method for assessing early endothelial dysfunction (7, 8). Combining biomarkers and non-invasive vascular examination techniques can provide more accurate assessment of endothelial status and guidance for intervention timing through the three dimensions of mechanism-function-molecule, while simultaneously breaking through the traditional lag model of waiting for structural damage before intervention.

Hypertension and obstructive sleep apnea-hypopnea syndrome (OSAHS) are significant risk factors for cardiovascular diseases. The prevalence of hypertension among adults in China has reached 27.9% (14), while the incidence of OSAHS is 14% in adult males and 5% in adult females (15). The coexistence of hypertension and OSAHS is reported to range from 30% to 50%, and the prevalence of hypertension in individuals with OSAHS is 56% (16, 17). Endothelial dysfunction is the foundation for the development of cardiovascular diseases. Hypertension and OSAHS frequently coexist. The individual detrimental impact of either hypertension or OSAHS on endothelial function is already well documented. It is reasonable to postulate that their combination activates synergistic injurious pathways that impose an additive, or even multiplicative, insult on the vascular endothelium. When both conditions are present, endothelial dysfunction is more pronounced than in either disease alone. To date, only Yuanyuan Xu et al. have specifically addressed this issue: in a cross-sectional analysis of 81 hypertensive patients without OSA (control group) and 62 hypertensive patients with moderate-to-severe OSA (OSA group), they demonstrated that the latter cohort exhibited both impaired endothelial function and adverse cardiac remodeling (18). The lack of clinical studies for stratifying endothelial dysfunction by the severity of concomitant hypertension and OSAHS means that clinicians lack the evidence to accurately assess disease burden or optimize intervention strategies in this high-risk population.

This study selected endothelial damage-related markers that are widely recognized in the literature and the RHI (Reactive Hyperemia Index), which is approved by the U.S. FDA, as the primary indicators to assess endothelial damage. In hypertensive patients with or without OSAHS (obstructive sleep apnea-hypopnea syndrome), a cross-sectional study was conducted to clarify the changes in RHI, vWF, VEGF, and EMPS levels in patients with various grades of hypertension combined with various severities of OSAHS.

## 2 Methods

### 2.1 Study participants

Hypertensive patients aged 35–60 years with concomitant OSAHS were selected.

[Note: The diagnostic criteria for hypertension are systolic blood pressure ≥140 mmHg and/or diastolic blood pressure ≥90 mmHg on three separate occasions. Patients who have been previously diagnosed and are taking antihypertensive medications are also included. The diagnostic criteria for OSAHS are as follows: 5 ≤ AHI < 15 (mild OSAHS), 15 ≤ AHI < 30 (moderate OSAHS), and AHI ≥ 30 (severe OSAHS).]

### 2.2 Exclusion criteria

1. Presence of diseases that would interfere with the monitoring of digital arterial pulsation, such as peripheral vascular disease, peripheral neuropathy, or finger deformities;
2. Use of nitrate medications or *α*-receptor blockers;
3. Bilateral cervicothoracic sympathectomy;
4. Consumption of alcohol or coffee, or use of sedatives on the day of the examination;
5. Inability to cooperate during the examination;
6. Pregnancy;
7. Other diseases that may affect the assessment of vascular endothelial function: secondary hypertension, diabetes mellitus, hypertensive emergency, acute coronary syndrome, acute stroke, chronic kidney disease stage 4 (CKD-4), acute heart failure, or severe diseases involving other systems;
8. Failure to sign the informed consent form.
9. If the study subject is unwilling to continue with any part of the examination and further communication proves ineffective, the signed informed consent form will be withdrawn.

### 2.3 Enrollment strategy

Patients from the Cardiology Outpatient Department of Chengdu Pixiu District People's Hospital were enrolled consecutively from July 1, 2023, to December 31, 2023.

### 2.4 Informed consent

This study is primarily non-invasive. However, during the detection of vWF and other parameters, a percutaneous venous blood draw of 3 mL is required, which may cause discomfort or pain to the patient. Patients need to be informed that the data obtained from the tests can provide guidance for the prevention of cardiovascular diseases. Most patients are able to understand and accept this. Since this study involves invasive procedures, written notification is required, and patients must sign an informed consent form.

### 2.5 Assessment of eligible subjects

For patients who meet the inclusion criteria, effective communication will be conducted. If the patient agrees, they will be enrolled. If the patient hesitates or refuses, they will not be enrolled.

### 2.6 Baseline data collection

Design of Baseline Data Form: The baseline data form mainly includes the following information: name, gender, age, phone number, and data entry number.

Design of Study Data Form: The study data form includes the following information: participant ID, gender, age, blood pressure, heart rate, presence of snoring, smoking and alcohol consumption status, cholesterol (CHOL), low-density lipoprotein cholesterol (LDL), fasting blood glucose (FBG), blood urea nitrogen, creatinine, urinary microalbumin, medication use (nitrate medications, *α*-receptor blockers, sedatives), coffee and alcohol consumption on the day of examination, history of diseases such as coronary heart disease, diabetes mellitus, hypertensive emergency, and whether the participant is pregnant.

### 2.7 Intervention measures

None.

### 2.8 Measurement method

#### 2.8.1 Measurement method of the endoPAT-2000 non-invasive vascular endothelial function detection system

The EndoPAT-2000 non-invasive vascular endothelial function detection system (manufactured by Itamar Medical Inc., Israel) is used to measure the reactive hyperemia index (RHI), an index of endothelium-dependent vasodilation. The specific operating method is referenced from the literature (6–8). Participants lie supine on the examination bed and rest for 30 minutes with their arms placed at their sides. An inflatable cuff is placed on the non-dominant upper arm, and two Endo-PAT probe finger cuffs are fitted on the index fingers of both hands without touching each other (the probes contain sensors and inflation devices). After turning on the Endo-PAT device, the probe finger cuffs are automatically inflated via the connecting tubes. The sensors within the probes transmit the blood flow signals from the digital arterial vascular bed to the computer software via the Endo-PAT system. Once the blood flow in the digital vascular bed stabilizes, a 15-minute endothelial function test begins. Initially, a 5-minute baseline recording of the digital vascular blood flow is obtained. Subsequently, the cuff is rapidly inflated to a pressure greater than 200 mmHg. When the Endo-PAT software indicates that the blood flow signal on one side has disappeared, this is recorded for 5 minutes. The cuff is then rapidly deflated to zero pressure, and the restored blood flow signal is recorded for another 5 minutes after deflation. The test is concluded by calculating the reactive hyperemia index (RHI) using the device's dedicated software to assess endothelial function.

#### 2.8.2 Measurement method of the watch-Pat200 portable sleep apnea monitoring system

The Watch-PAT200 (manufactured by Itamar Medical Ltd.) is a portable sleep monitor that assesses respiratory events by detecting changes in sympathetic activity. It reflects the body's sympathetic activity through the measurement of changes in the volume of the finger's distal artery, and thus determines respiratory events. Specifically, when a respiratory event occurs, sympathetic activity increases, leading to the constriction of the finger artery and a decrease in the PAT (Peripheral Arterial Tone) signal. The report is automatically generated by the device's software system. This device is the only portable monitoring device recommended for the diagnosis of obstructive sleep apnea in adults in the 2016 AASM (American Academy of Sleep Medicine) Clinical Practice Guidelines (19). In this study, the main parameter monitored is the respiratory disturbance index—the Apnea-Hypopnea Index (AHI), with a recording duration of no less than 7 hours.

#### 2.8.3 Laboratory tests

vWF, VEGF and EMPS was measured using the ELISA method. The assay was performed using a sandwich enzyme-linked immunosorbent assay (ELISA) kit. The target antibody was pre-coated onto a 96-well microplate to form a solid-phase carrier. Standards or samples were then added to the microplate wells, allowing the target within them to bind to the immobilized antibody. Following this, a horseradish peroxidase (HRP)-labeled detection antibody was added. After washing away any unbound substances, a 3,3’,5,5'-tetramethylbenzidine (TMB) substrate solution was added for color development. TMB is catalyzed by the peroxidase to produce a blue color, which finally turns yellow after the addition of a stop solution (acid). The intensity of the developed yellow color is directly proportional to the concentration of the target present in the sample. The optical density (OD) was measured at a wavelength of 450 nm using a microplate reader, and the sample concentration was calculated accordingly. The following ELISA kits from ZCIBIO Technology Co.,Ltd (Shanghai, China) were employed:

Human Endothelial Microparticles (EMPs) ELISA Kit (96 tests; Cat# ZC-56542)

Human Vascular Endothelial Growth Factor (VEGF) ELISA Kit (96 tests; Cat# ZC-35248)

Human von Willebrand Factor (vWF) ELISA Kit (96 tests; Cat# ZC-35286).

#### 2.8.4 Blinding of some investigators

After completing the EndoPAT-2000 measurement, participants provided baseline information and blood samples prior to undergoing the Watch-PAT200 assessment on the same day. EndoPAT-2000 and Watch-PAT200 tests were conducted in an open-label manner: The test results were automatically generated. For the detection of von Willebrand Factor (vWF), Vascular Endothelial Growth Factor (VEGF), and Endothelial Microparticles (EMPs), manual operations were performed by a specialized laboratory technician who was proficient in the Elisa method and flow cytometry (the only designated person for this study). However, the laboratory technician was blinded to the basic information of each participant.

#### 2.8.5 Follow-up plan

No, only vascular health guidance is provided to the study subjects.

### 2.9 Statistical methods

In this study, all statistical analyses were performed using R version 4.2.3, RStudio, and the dplyr and corrgram packages. All statistical tests were two-sided, with a significance level set at *α* = 0.05.

Based on the clinical data previously collected by the authors, the mean RHI of the experimental group (hypertension combined with severe OSAHS) was roughly estimated to be 1.31 with a standard deviation of 0.14, while that of the control group (hypertension combined with mild OSAHS) was 1.5 with a standard deviation of 0.3. The significance level (α) was set at 0.05, with a type II error rate (β) of 0.20 and a statistical power (1-*β*) of 0.80.With a 1:1 allocation ratio, the total sample size was calculated to be 48 participants.

Prior to data analysis, suspicious outliers were identified with box plots; values lying outside Q1−1.5 × IQR over Q3 + 1.5 × IQR were defined as outliers and excluded from statistical analysis. Continuous variables were tested for normality; normally distributed data are described as mean ± SD, whereas non-normally distributed data are described as median (Q1—Q3).

## 3 Results

### 3.1 Descriptive statistical results

Table 1 presents the means (Mean) and standard deviations (SD) of various physiological and biochemical indicators for subjects in different hypertension categories (Grade 1, Grade 2, and Grade 3), along with the corresponding *P*-values. The results show that there is a significant difference in body mass index (BMI) among the groups (*P* = 0.047), with BMI being significantly higher in the Grade 2 and Grade 3 hypertension groups compared to the Grade 1 group. Although the P-values for other indicators such as height, age, and heart rate did not reach statistical significance, age was notably higher in the Grade 2 hypertension group (51.00 ± 6.97 years), suggesting a potential relationship between hypertension grade and age. Other indicators such as systolic blood pressure, diastolic blood pressure, and biochemical markers (e.g., cholesterol, proteinuria) did not show significant differences. Overall, BMI appears to be an important factor influencing the severity of hypertension, while other physiological indicators did not show clear group differences

**Table 1:** Descriptive statistics for continuous variables.

Variable	Total	Grade 1 hypertension	Grade 2 hypertension	Grade 3 hypertension	*P-*value
BH (Mean ± SD)	1.68 ± 0.06	1.70 ± 0.06	1.67 ± 0.08	1.69 ± 0.05	0.571
BW(Mean ± SD)	81.22 ± 12.46	74.00 ± 15.21	80.83 ± 9.93	83.54 ± 12.21	0.164
BMI(Mean ± SD)	28.65 ± 3.88	25.61 ± 4.26	29.04 ± 2.98	29.37 ± 3.81	0.047
Age(Mean ± SD)	47.55 ± 8.05	42.25 ± 9.74	51.00 ± 6.97	47.59 ± 7.39	0.055
SBP(Mean ± SD)	144.64 ± 22.30	136.88 ± 11.24	147.25 ± 17.51	145.78 ± 26.33	0.558
DBP(Mean ± SD)	97.04 ± 16.30	90.25 ± 10.99	96.33 ± 13.64	99.37 ± 18.40	0.383
HR(Mean ± SD)	85.00 ± 13.99	79.62 ± 7.91	90.92 ± 13.76	83.96 ± 14.94	0.178
CHOL(Mean ± SD)	4.87 ± 0.91	4.86 ± 0.67	5.15 ± 0.98	4.75 ± 0.94	0.457
LDL(Mean ± SD)	3.34 ± 0.96	3.52 ± 0.98	3.37 ± 0.76	3.28 ± 1.06	0.820
FBG(Mean ± SD)	6.62 ± 7.24	4.65 ± 0.87	9.21 ± 13.63	6.06 ± 3.07	0.326
BUN(Mean ± SD)	4.89 ± 1.18	4.56 ± 0.74	5.03 ± 1.47	4.93 ± 1.17	0.667
SCr(Mean ± SD)	76.51 ± 13.88	73.41 ± 7.19	76.97 ± 13.73	77.22 ± 15.61	0.793
UmA(Mean ± SD)	48.14 ± 62.82	20.14 ± 14.06	48.13 ± 58.69	56.44 ± 71.82	0.365
AHI(Mean ± SD)	32.18 ± 20.00	28.25 ± 9.61	33.19 ± 25.31	32.89 ± 20.15	0.836
RHI(Mean ± SD)	1.60 ± 0.46	1.49 ± 0.15	1.73 ± 0.70	1.58 ± 0.39	0.512
vWF(Mean ± SD)	20.74 ± 9.68	24.40 ± 6.06	17.95 ± 8.98	20.90 ± 10.68	0.350
VEGF(Mean ± SD)	395.53 ± 181.59	465.91 ± 123.35	336.58 ± 145.86	400.88 ± 205.14	0.294
EMPS(Mean ± SD)	321.77 ± 138.73	349.67 ± 82.69	287.69 ± 144.20	328.65 ± 150.29	0.583

BH, body BH (m); BW, body BW (Kg); Age, Age (years old); BMI, body mass index (Kg/m^2^); SBP, systolic_pressure (mmHg); DBP, diastolic_pressure (mmHg); HR, heart rate (bpm); CHOL, cholesterol (mmol/L); LDL, low density lipoprotein cholesterol (mmol/L); FBG, fasting blood glucose (mmol/L); BUN, blood urea nitrogen (mmol/L); SCr, serum creatinine (umol/L); UmA, urinary microalbumin (mg/L); AHI, apnea hypopnea index (times/Hour); RHI, reactive hyperemia index; vWF, von Willebrand factor (ng/mL); VEGF, vascular endothelial growth factor (pg/mL); EMPs, endothelial microparticles (pg/mL).

### 3.2 Descriptive statistics for categorical variables

Table 2 presents the descriptive statistics of categorical variables for subjects in different hypertension categories (Grade 1, Grade 2, and Grade 3), including risk level, weight status, gender, Coronary Atherosclerotic Disease, and lifestyle habits. The results show that the proportion of low-risk individuals in Grade 1 hypertension is significantly higher (*P* = 0.003), while the proportion of Grade 1 hypertension in the high-risk group is also relatively high (62.5%). Additionally, the proportion of males in Grade 3 hypertension is 100% (*P* = 0.042), indicating that males are predominant in Grade 3 hypertension. Although the proportions of obesity and OSA (obstructive sleep apnea) vary among the groups, no statistical significance was observed (all *P*-values > 0.05). Overall, low risk, high risk, and gender are significantly associated with hypertension grade, while other lifestyle factors did not show significant differences.

**Table 2:** Descriptive statistics for categorical variables.

Variable	Total	Grade 1 hypertension	Grade 2 hypertension	Grade 3 hypertension	*P-*value
Low-risk	1 (2.1)	1 (12.5)	0 (0.0)	0 (0.0)	0.003
High-risk	10 (21.3)	5 (62.5)	2 (16.7)	3 (11.1)	
Very high-risk	36 (76.6)	2 (25.0)	10 (83.3)	24 (88.9)	
Ideal body weight	4 (8.5)	2 (25.0)	0 (0.0)	2 (7.4)	0.251
Overweight	17 (36.2)	3 (37.5)	6 (50.0)	8 (29.6)	
Obesity	26 (55.3)	3 (37.5)	6 (50.0)	17 (63.0)	
Mild OSAHS	14 (29.8)	1 (12.5)	5 (41.7)	8 (29.6)	0.486
Moderate OSAHS	9 (19.1)	3 (37.5)	2 (16.7)	4 (14.8)	
Severe OSAHS	24 (51.1)	4 (50.0)	5 (41.7)	15 (55.6)	
Male	43 (91.5)	6 (75.0)	10 (83.3)	27 (100.0)	0.042
Female	4 (8.5)	2 (25.0)	2 (16.7)	0 (0.0)	
Hypertension	3 (6.4)	2 (25.0)	0 (0.0)	1 (3.7)	0.056
Hypertension + OSAHS	44 (93.6)	6 (75.0)	12 (100.0)	26 (96.3)	
Non-CAD	37 (78.7)	7 (87.5)	9 (75.0)	21 (77.8)	0.786
CAD	10 (21.3)	1 (12.5)	3 (25.0)	6 (22.2)	
Non-smoking	21 (44.7)	5 (62.5)	6 (50.0)	10 (37.0)	0.406
Smoking	26 (55.3)	3 (37.5)	6 (50.0)	17 (63.0)	
Non-drinking	37 (78.7)	5 (62.5)	9 (75.0)	23 (85.2)	0.362
Drinking	10 (21.3)	3 (37.5)	3 (25.0)	4 (14.8)	
IGT	3 (6.4)	0 (0.0)	1 (8.3)	2 (7.4)	0.357
Diabetes mellitus	11 (23.4)	0 (0.0)	4 (33.3)	7 (25.9)	

OSAHS, obstructive sleep apnea-hypopnea syndrome; CAD, coronary atherosclerotic disease; IGT, impaired glucose tolerance.

### 3.3 Analysis of correlations for main variables

#### 3.3.1 Table of correlation analysis for main variables

Table 3 shows the correlations among multiple variables, including a significant positive correlation between height and weight (r = 0.471, *P* = 0.001), a strong positive correlation between weight and BMI (r = 0.887, *P* < 0.001), and a significant negative correlation between age and diastolic blood pressure (r = −0.329, *P* = 0.024). There is also a strong positive correlation between urinary microalbumin and AHI (r = 0.514, *P* < 0.001). A strong negative correlation exists between AHI and RHI (r = −0.579, *P* = 0.001).

**Table 3:** Correlation analysis table.

Variable 1	Variable 2	Correlation coefficient	*P*-value
BH	BW	0.471	0.001
BW	BMI	0.887	0.000
BMI	UmA	0.292	0.046
Age	DBP	−0.329	0.024
Age	BUN	0.404	0.005
Age	AHI	0.347	0.017
Age	VEGF	0.296	0.043
Age	EMPS	0.327	0.025
SBP	DBP	0.817	<0.001
DBP	HR	0.408	0.004
DBP	RHI	0.267	0.070
DBP	VWF	−0.307	0.036
DBP	VEGF	−0.316	0.031
DBP	EMPS	−0.353	0.015
HR	CHOL	0.303	0.038
HR	VWF	−0.444	0.002
HR	VEGF	−0.430	0.003
HR	EMPS	−0.498	<0.001
CHOL	LDL	0.717	<0.001
CHOL	BUN	0.325	0.026
CHOL	SCr	0.408	0.004
LDL	SCr	0.291	0.047
FBG	UmA	0.375	0.010
BUN	SCr	0.392	0.006
UmA	AHI	0.514	<0.001
UmA	VEGF	0.293	0.046
AHI	RHI	−0.579	<0.001
AHI	VWF	0.558	<0.001
AHI	VEGF	0.488	0.001
AHI	EMPS	0.655	<0.001
RHI	VWF	−0.624	<0.001
RHI	VEGF	−0.625	<0.001
RHI	EMPS	−0.673	<0.001
VWF	VEGF	0.887	<0.001
VWF	EMPS	0.945	<0.001
VEGF	EMPS	0.901	<0.001

BH, body BH (m); BW, body BW (Kg); Age, Age (years old); BMI, body mass index (Kg/m^2^); SBP, Systolic_pressure (mmHg); DBP, diastolic_pressure (mmHg); HR, heart rate(bpm); CHOL, cholesterol (mmol/L); LDL, low density lipoprotein cholesterol (mmol/L); FBG, fasting blood glucose (mmol/L); BUN, blood urea nitrogen (mmol/L); SCr, serum creatinine (umol/L); UmA, urinary microalbumin (mg/L); AHI, apnea hypopnea index (times/hour); RHI, reactive hyperemia index; VWF, von Willebrand factor (ng/mL); VEGF, vascular endothelial growth factor (pg/mL); EMPS, endothelial microparticles (pg/mL).

#### 3.3.2 Correlation analysis chart for main continuous variables

**Figure 1:**
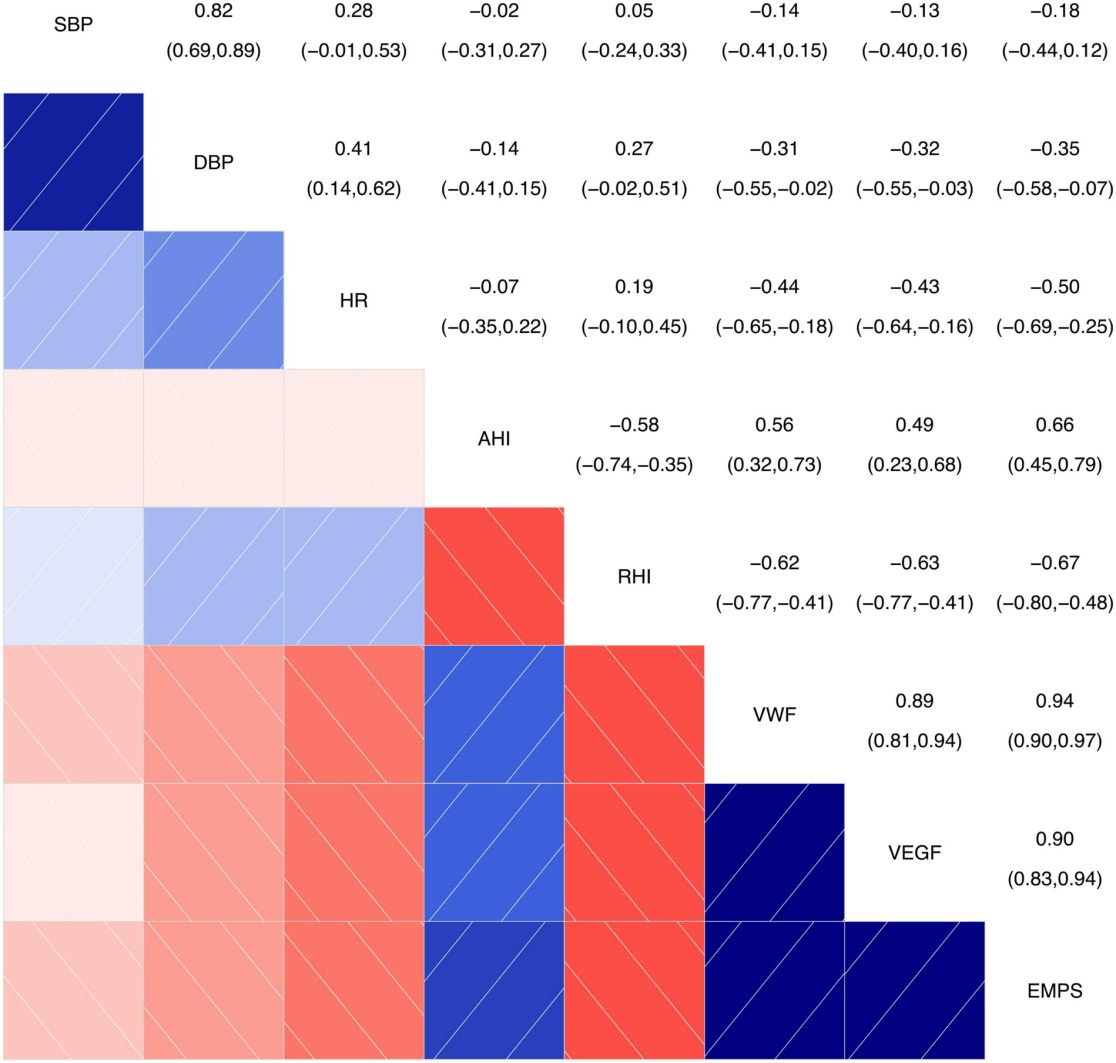
Correlation analysis chart for main continuous variables. SBP, systolic blood pressure; DBP, diastolic blood pressure; HR, heart rate; AHI, apnea-hyponea index; RHI, reactive hyperemia index; VWF, von Willebrand factor; VEGF, vascular endothelial growth factor; EMPS, emicroparticles. Color and Correlation: Blue indicates a positive correlation, with darker shades of blue representing stronger correlations. Red indicates a negative correlation, with darker shades of red representing stronger negative correlations. White or light-colored areas indicate weak or near-zero correlations. Numerical Interpretation: The numbers range from the top-left to the bottom-right corner. The diagonal shows the correlation of each variable with itself, which is always 1. The other numbers represent the correlation coefficients between two variables.

As shown in Figure 1, the intersection between AHI and RHI shows a correlation coefficient of −0.58 (−0.75, −0.35), indicating a certain degree of negative correlation between these two variables. The intersections between AHI and vWF, VEGF, and EMPs show correlation coefficients of 0.56 (0.32, 0.73), 0.49 (0.23, 0.68), and 0.66 (0.45, 0.79), respectively, indicating a certain degree of positive correlation between AHI and the variables (vWF, VEGF, and EMPs), with *P* ≤ 0.001.

The intersections between RHI and vWF, VEGF, and EMPs show correlation coefficients of −0.62 (−0.77, −0.41), −0.63 (−0.77, −0.41), and −0.67 (−0.80, −0.48), respectively, indicating a certain degree of negative correlation between RHI and the variables (vWF, VEGF, and EMPs), with *P* < 0.001.

#### 3.3.3 Correlation analysis chart of categorical ordinal variables: hypertension stage, grade, and correlations with AHI and RHI

**Figure 2:**
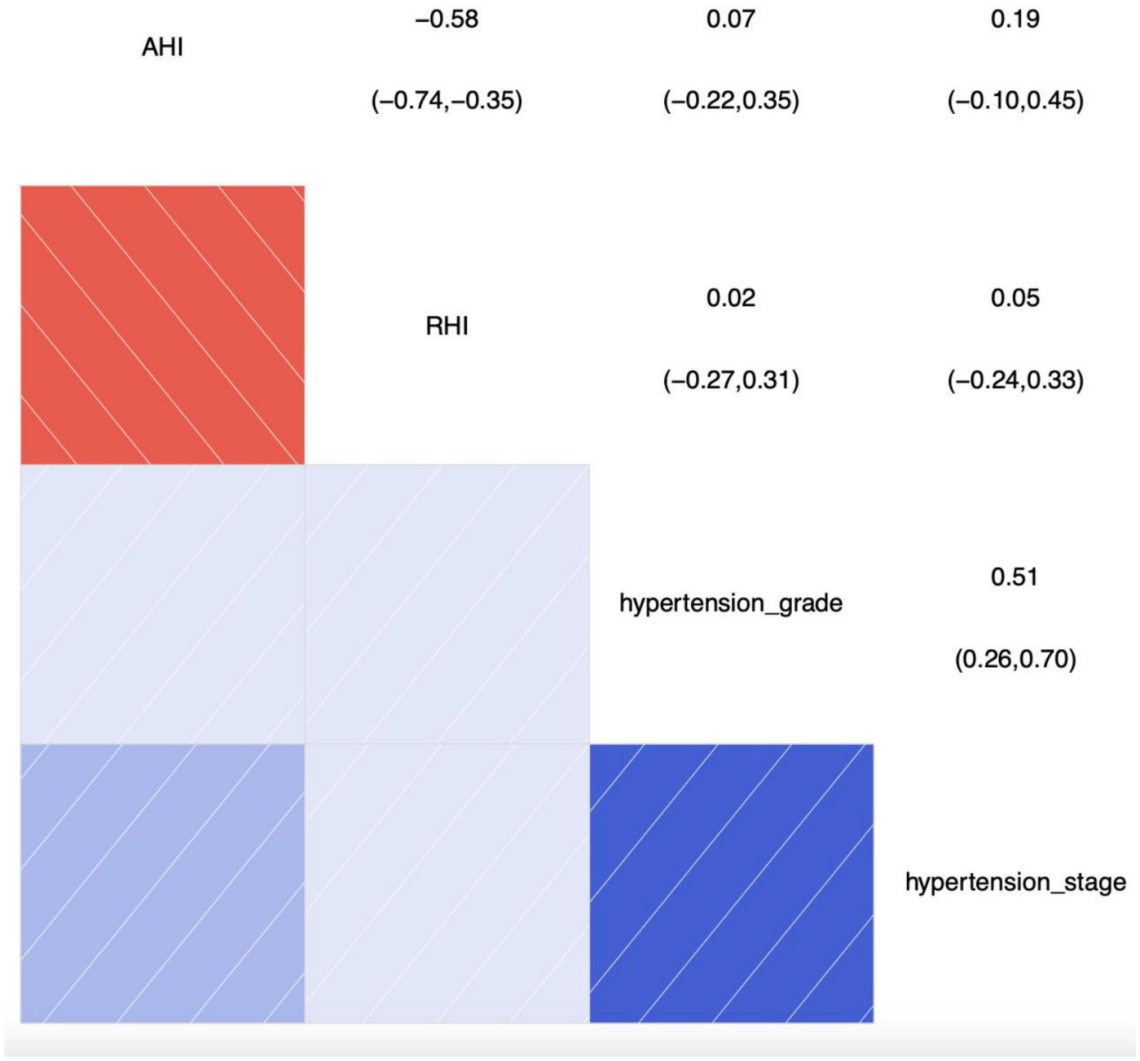
Correlation analysis chart of categorical ordinal variables: hypertension stage, grade, and correlations with AHI and RHI. SBP, systolic blood pressure; AHI, apnea-hyponea index; RHI, reactive hyperemia index; Color and Correlation: Blue indicates a positive correlation, with darker shades of blue representing stronger correlations. Red indicates a negative correlation, with darker shades of red representing stronger negative correlations. White or light-colored areas indicate weak or near-zero correlations. Numerical Interpretation: The numbers range from the top-left to the bottom-right corner. The diagonal shows the correlation of each variable with itself, which is always 1. The other numbers represent the correlation coefficients between two variables.

Figure 2 shows the results of the correlation analysis between AHI, RHI, and hypertension grade and hypertension stage, with correlation coefficients less than 0.2, indicating that there is almost no linear relationship between them. This further indicates that the correlations between AHI, RHI, and hypertension grade and hypertension stage are not significant. Therefore, these results suggest that AHI and RHI may not significantly influence hypertension grade and hypertension stage, or that the associations between hypertension grade and hypertension stage and AHI and RHI are very weak.

#### 3.3.4 Linear regression analysis of RHI with primary indicators

As shown in Table 4, the relationships of AHI, endothelial damage-related markers, hypertension grade, and stratification with RHI were analyzed by linear regression analysis and multiple linear regression analysis(adjustment for BMI, age, smoking, alcohol and LDL). The RHI showed significant inverse associations with the studied variables. A 1-SD increase in AHI was associated with a decrease in RHI of 0.02 units (*β* = –0.02, adjusted *β* = –0.66, *P* < 0.01). A 1-SD increase in vWF was associated with a decrease in RHI of 0.62 units (*β* = –0.62, adjusted *β* = –0.63, *P* < 0.01). A 1-SD increase in VEGF was associated with a decrease in RHI of 0.63 units (*β* = −0.63, adjusted *β* = –0.62, *P* < 0.01). A 1-SD increase in EMPs was associated with a decrease in RHI of 0.67 units (*β* = −0.67, adjusted *β* = –0.70, *P* < 0.01). AHI, vWF, VEGF, and EMPs may be identified as independent risk factors for RHI, and these associations remained significant after adjustment for BMI and age. Hypertension grade and stage are not independent risk factors for RHI.

**Table 4:** Linear regression analysis of RHI with primary indicators.

Independent variable	*β*	*P*	Adjusted *β*	Adjusted 95%Cl		*P*
				Upper limit	Lower limit	
AHI	−0.02	<0.01	−0.66	0.02	0.01	<0.01
vWF	−0.62	<0.01	−0.63	−0.04	−0.02	<0.01
VEGF	−0.63	<0.01	−0.62	0.00	−0.01	<0.01
EMPs	−0.67	<0.01	−0.70	−0.003	−0.002	<0.01
Hypertension grade	0.01	0.99	–	–	–	–
Hypertension stage	0.09	0.54	–	–	–	–

AHI, apnea hypopnea index (times/hour); RHI, reactive hyperemia index; VWF, von Willebrand factor (ng/mL); VEGF, vascular endothelial growth factor (pg/mL); EMPS, endothelial microparticles (pg/mL).

## 4 Discussion

Patients with hypertension combined with OSAHS (Obstructive Sleep Apnea-Hypopnea Syndrome) are clinically common, but patients often neglect the treatment of OSAHS, leading to the occurrence of cardiovascular and cerebrovascular diseases, and even sudden death. The use of vWF to detect vascular endothelial function in patients with hypertension and OSAHS has been widely reported both domestically and internationally (20, 21), and it is recognized as a highly reliable marker of vascular endothelial damage (22). It can accurately assess vascular endothelial health status, and vWF levels can return to normal following antihypertensive treatment. There are relatively few reports on the use of RHI (Reactive Hyperemia Index) to detect vascular endothelial dysfunction in patients with hypertension combined with OSAHS, mainly reflecting the correlation between RHI and other related factors before and after treatment, such as acute cerebral infarction, renal dialysis, coronary heart disease, hypertension, and OSAHS (23, 24). Simultaneous use of vWF and RHI to evaluate vascular endothelial health status in patients with heart failure and peripheral vascular disease has also been reported (25, 26), but there are fewer studies on hypertension combined with OSAHS. In China, five studies have examined the correlation between hypertension, RHI, and vascular endothelial function (27, 28), but none have specifically addressed the impact of coexistent hypertension and OSAHS on RHI and endothelial damage-related markers. This study may preliminarily address this gap.

In the relationship between AHI and other health indicators, the study found a significant positive correlation between AHI and endothelial damage-related markers (vWF, VEGF and EMPs). This suggests that an increase in AHI levels (indicating greater severity of obstructive sleep apnea-hypopnea syndrome) may be associated with changes in vascular health, thereby further affecting cardiovascular endothelial function. These findings are consistent with the reports by Micha Harańczyk and Peker Y et al. (29, 30).

Beside of the endothelial damage-related markers, in individuals with hypertension combined with obstructive sleep apnea-hypopnea syndrome (OSAHS), a reduced RHI suggests a potential increase in the risk of cardiovascular and cerebrovascular diseases in the future. There is a negative correlation between AHI and RHI. The cross-sectional analysis of “AHI” and “RHI” showed a correlation coefficient of −0.58 (−0.75, −0.35), indicating a significant negative correlation between these two variables (*P* < 0.001). AHI may be an independent risk factors of RHI (*β* = –0.02, adjusted *β* = –0.60, *P* < 0.01). Each 1-standard deviation increase in AHI was associated with a 0.02-unit decrease in RHI. The correlation between AHI and RHI in our study is similar to that reported by Dorota Ochijewicz (31). This indicates that the severity of sleep apnea is significantly associated with an increased likelihood of vascular endothelial dysfunction. If OSAHS is not corrected, it portends an increased risk of cardiovascular and cerebrovascular diseases (32). However, as this study is a cross-sectional design, further prospective studies are needed to elucidate the relationship between AHI and RHI.

RHI showed a significant negative correlation with endothelial damage-related markers. This indicates that a decrease in vascular endothelial response, as measured by RHI, may be accompanied by an increase in endothelial damage-related markers.This finding reveals the correlation between non-invasive vascular endothelial function indicators (RHI) and endothelial damage-related markers. As a non-invasive detection indicator, RHI holds greater advantages in serving as both an “early warning system” and a “therapeutic navigator” for cardiovascular risk. This integrated assessment model is of paramount clinical significance in facilitating early intervention and reducing long-term cardiovascular risk.

In the correlation analysis between RHI and endothelial damage-related markers, EMPs emerged as the strongest driver of RHI values. Although vWF and VEGF are established markers of endothelial dysfunction, they chiefly mirror endothelial activation or functional modulation rather than direct cellular damage; their elevations are additionally confounded by inflammation, coagulation activation and hypoxia-driven compensatory pathways. EMPs, membrane vesicles released during endothelial apoptosis, activation or overt injury, directly document loss of structural integrity. Being a “direct product” of damaged endothelial cells, EMPs show a closer pathobiological correspondence with RHI, a functional measure of endothelium-dependent dilation, and therefore exhibit the tighter correlation.

This study has identified several significant variable relationships, providing important clues for understanding the potential associations between health indicators. The correlation between age and multiple health indicators is significant. In particular, positive correlations were observed between age and blood urea nitrogen (BUN), AHI, VEGF, VWF, and EMPS, suggesting that aging has an impact on renal function, respiratory status, and vascular health. This is consistent with the normal human aging process.

The study found that patients with obstructive sleep apnea-hypopnea syndrome (OSAHS) have increased microalbuminuria, indicating impaired vascular endothelial function (33), which is positively correlated with AHI (34) and negatively correlated with RHI. There are also positive correlations with endothelial damage-related markers, although these correlations do not show significant statistical significance. There is a need to expand the study sample size.

The correlation between AHI, RHI, hypertension grade, and hypertension stage is weak, failing to demonstrate a significant linear relationship. This result may be related to limitations in sample size or may indicate a more complex nonlinear relationship between AHI and RHI and hypertension grade and hypertension stage.

It is also possible that, although hypertension grade and hypertension stage represent higher—risk patients, effective blood pressure control and management of hypertension risk factors may have influenced the results. Specifically, when measuring RHI, VWF, VEGF, and EMPS, good control of blood pressure and other risk factors such as diabetes may have been achieved. As a result, there may be no obvious correlation between the level of AHI or RHI and hypertension grade and hypertension stage (35).

Thus, RHI can be used as a clinically meaningful tool at the stages of initial evaluation, etiologic screening, therapeutic decision-making, and longitudinal follow-up in hypertension.

At first diagnosis: an RHI < 1.67 identifies subjects with incipient endothelial dysfunction and refines cardiovascular risk stratification beyond office blood pressure. When RHI is markedly reduced: systematic screening for OSAHS is indicated; if confirmed, prompt CPAP therapy should be recommended.
Combined assessment: integrating RHI with plasma EMPs, vWF, or other endothelial biomarkers creates a “function + molecule” dual-axis profile that guides the need for intensive antihypertensive, anti-inflammatory, or antioxidant interventions.
Follow-up: repeat RHI measurement 3–6 months after CPAP, antihypertensive, or weight-loss therapy allows objective verification of endothelial recovery. Persistently low RHI: signals high residual cardiovascular risk and triggers further therapeutic escalation—e.g., stricter lipid control or targeted anti-inflammatory therapy.

Embedding RHI into routine care therefore enables a truly individualized management algorithm for hypertension.

This study has revealed significant associations between body weight, blood pressure, age, and a variety of health indicators, providing foundational data for further understanding of health risk factors. However, the correlations between some variables are not significant, indicating that there may be a need to increase the sample size in future studies, especially regarding the relationship between AHI and the grading of hypertension, to further explore potential non-linear associations and moderating factors. Future studies should consider multi-center designs, include normotensive controls, and adopt prospective cohorts to further clarify (1) the role of AHI in the onset and progression of hypertension and (2) how advancing hypertension influences vascular endothelial function indices such as RHI. These findings provide an important basis for health monitoring, disease prevention, and the development of intervention strategies, and they also offer direction for future in-depth research into the causal relationships between these variables.

## 5 Conclusion

Through the correlation analysis in this study, among patients with hypertension combined with OSAHS, AHI is negatively correlated with RHI, and positively correlated with the endothelial damage-related markers. In patients with hypertension combined with OSAHS, RHI can be used as an important indicator in routine tests of vascular endothelial function to predict the degree of vascular endothelial injury. Emphasis on OSAHS treatment is needed.

RHI is negatively correlated with these endothelial damage-related markers. The increase in endothelial damage-related markers is associated with a decrease in RHI.As a non-invasive detection indicator, RHI holds greater advantages in serving as both an “early warning system” and a “therapeutic navigator” for cardiovascular risk. This integrated assessment model is of paramount clinical significance in facilitating early intervention and reducing long-term cardiovascular risk. Embedding RHI into routine care therefore enables a truly individualized management algorithm for hypertension.

The correlation analysis between AHI, RHI and hypertension grade and hypertension stage showed that the correlations between AHI, RHI and hypertension grade and hypertension stage were weak and not significant. Although the current sample did not provide evidence that AHI significantly affects hypertension grade or hypertension stage, nor that hypertension grade or stage has an effect on RHI. Future studies can consider multi-center designs, include normotensive controls, and adopt prospective cohorts to further clarify the role of AHI in the onset and progression of hypertension and how advancing hypertension influences vascular endothelial function indices such as RHI.

Overall, the findings of this study provide preliminary insights into the relationships between the variables. Future research should expand the sample size, particularly in the analysis of AHI and hypertension grade and hypertension stage, and introduce stratified and multivariate models to provide more scientifically based guidance for clinical interventions.

## Data Availability

The original contributions presented in the study are included in the article/Supplementary Material, further inquiries can be directed to the corresponding authors.
